# The “Ets” Factor: Vessel Formation in Zebrafish—The Missing Link?

**DOI:** 10.1371/journal.pbio.0040024

**Published:** 2006-01-17

**Authors:** Lucy J Patterson, Roger Patient

## Abstract

The zebrafish offers a powerful model for studying the development of new blood vessels.

As the major conduit of oxygen and nutrients to cells and tissues, blood vessels are the body's ultimate lifeline (Figure 1). From conception, embryos must perform all the necessary functions of life before they have developed the organs to do so. The raw materials and waste products of metabolism are initially exchanged through simple diffusion, but as the embryo grows larger and more complicated, this is no longer sufficient. Consequently, the cardiovascular system is the first functional organ to form in the developing embryo.

**Figure 1:**
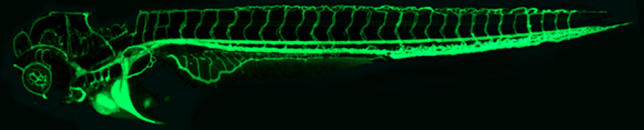
The Fine Detail of the Developing Vasculature of an approximately four-day-old Zebrafish Embryo Is Revealed Using Confocal Microangiography The accessibility and optical clarity of zebrafish embryos lend a particular advantage to the study of vascular development. Fluorescent microspheres can be injected into the blood stream, penetrating the entire patent vascular system, allowing imaging of the vasculature using confocal microscopy. (Photo: B. Weinstein)

New blood vessels are, in general, formed by two different processes. During vasculogenesis, new vessels arise from endothelial precursors (angioblasts) in the previously avascular early embryo. During angiogenesis, existing vessels undergo remodelling, and new vessels sprout from pre-existing ones. This latter process occurs both during development and in postnatal life. Although vasculogenesis and angiogenesis occur in different contexts, they are similar processes in molecular terms. During angiogenesis, mature endothelial cells must revert to an earlier, more plastic state in order to participate in the formation of new vessels. Therefore, many of the signals that initiate angiogenic remodelling, for example, vascular endothelial growth factor (VEGF), are also associated with vasculogenesis.

Deregulation of angiogenesis can contribute to the pathogenesis of a wide range of diseases, including cancer. Because tumours induce and depend on the growth of new blood vessels, factors that promote tumour angiogenesis are important targets for anticancer therapy. Given the molecular overlap between angiogenesis and vasculogenesis, a fuller understanding of vascular development in the early embryo is likely to provide valuable insight into potential therapeutic targets for treating vascular disease.

Blood and the vessels that transport it around the body emerge almost simultaneously, and in many cases, it is widely believed, from a common precursor. In this way, these two cell types, whose fates are so closely entwined, are specified at the same time and in the same place. This concept was first proposed by Florence Sabin early in the last century [1]. Studying the initial formation of blood and endothelial cells in the chick blastoderm, she observed how the two lineages arose from the same mesodermal tissue in the blood islands (an extraembryonic tissue surrounding the embryo on the surface of the yolk). The hypothesis was further advanced by P. D. F. Murray, who first coined the term “haemangioblast” (Figure 2) to describe the putative common precursor of the two lineages [2]. Ultrastructural studies of the mouse extraembryonic yolk sac revealed a similar close association between haematopoietic and endothelial development, and the concept of the haemangioblast was extended to mammalian development [3]. Since these early beginnings, many lines of evidence have accumulated to support the existence of the haemangioblast. Early haematopoietic and endothelial cells express many of the same genes, several of which are essential for the development of both lineages (reviewed in [4]). Similarly, a specific, although as yet uncharacterised, mutation in zebrafish, known as cloche, produces embryos with profound defects in both haematopoietic and endothelial lineages [5].

**Figure 2:**
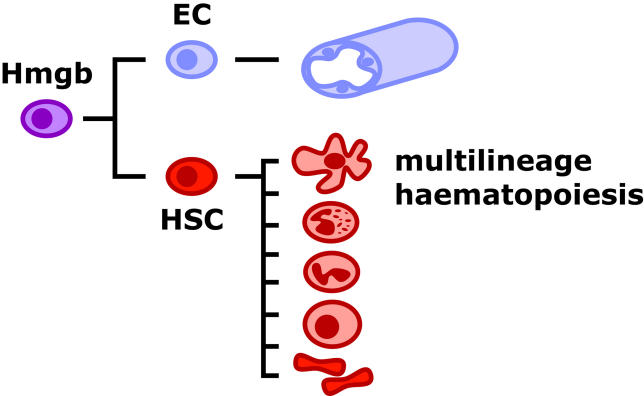
The Haemangioblast Many lines of evidence support the notion that blood and endothelium share a common precursor, the haemangioblast. EC, endothelial cell; Hmgb, haemangioblast; HSC, haematopoietic stem cell.

The most direct evidence for the existence of the haemangioblast comes from studies using a model system based on the in vitro differentiation of pluripotent mouse embryonic stem cells. In appropriate culture conditions, these cells spontaneously differentiate, forming colonies of cells, known as embryoid bodies, comprising a wide mix of cell types. The distribution and timing of the appearance of specific cell types, including haematopoietic and endothelial cells, in these colonies essentially recapitulates that of the developing embryo. Using this system, individual cells derived from embryoid bodies cultured in the presence of VEGF were identified that produced colonies of haematopoietic and endothelial cells [6]. These cells are termed blast-colony forming cells, and represent a transient population that develops prior to the emergence of identifiable haematopoietic cells during embryoid body differentiation. For these reasons, the blast-colony forming cell is thought of as the in vitro equivalent of the haemangioblast.

Recently, comparable progenitor cells have been isolated from early mouse embryos, which form bilineage colonies under similar culture conditions [7]. These cells are the closest yet to a genuine in vivo haemangioblast, but their haematopoietic and endothelial potential has only been demonstrated in vitro. We cannot be sure that these cells would perform the same way if left in the embryonic environment. Thus, although there is considerable evidence for the haemangioblast's existence, definitive proof ultimately awaits an in vivo lineage labelling experiment showing that a single precursor cell can give rise to both haematopoietic and endothelial progeny.

## Making a Choice—Determining Endothelial Cell Fate

Once formed, the putative haemangioblast must make the choice between haematopoietic and endothelial cell fate. While the hierarchy of transcription factors that programme haematopoietic development, spearheaded by Scl, is relatively well characterised, those governing endothelial cell specification remain more elusive. However, some of the signalling pathways involved in the differentiation and proliferation of endothelial precursors and in their subsequent migration and patterning have been identified. Most notably, VEGF is one of the most critical and specific drivers of vascular development, known to be intimately involved in endothelial precursor specification and patterning. Onset of expression of the VEGF receptor *flk1*, along with *scl*, marks the earliest precursors of the developing endothelial lineage, both in the yolk sac and in the embryo proper [8]. In the yolk sac blood islands, these cells give rise to both blood and endothelium, and thus appear to be haemangioblasts. Cells coexpressing *flk1* and *scl* generated in vitro in embryoid bodies also give rise to both lineages [9]. Despite its early associations, *flk1* expression is later restricted to endothelial cells [8,10]. Recently VEGF signalling via Flk1 has been shown to be crucial for the allocation of early mesoderm to the endothelial lineage [11].

Insight into the role of VEGF signalling during vascular development has also been provided by gene-targeting experiments in the mouse. Mice generated with mutations in three different proteins—F*lk1^−/−^*, *VEGF^+/−^*, or *plcg1^−/−^* (a downstream effector of VEGF signalling)—die at midgestation (E8.5–E9.5) due to a complete lack of endothelial or haematopoietic development [12–15]. Further studies in both murine and amphibian embryos suggested that VEGF signalling is required for the correct migration of haemangioblast/angioblast precursors to their sites of differentiation [16,17]. However, the defects of the *flk1^−/−^* mouse cannot be ascribed to impaired migration alone, since development of angioblasts was completely blocked and the embryos failed to form even a primitive vascular plexus [12]. VEGF signalling is therefore essential for initial specification, proliferation, and migration of early vascular progenitors in mouse embryos.

In contrast to experiments in the mouse, disruption of the VEGF signalling pathway in zebrafish by way of mutation, antisense knockdown, or inhibition of ligand or receptors [18–21] does not produce the same severe early defects in vasculogenesis. Later defects in dorsal aorta specification and sprouting of intersomitic vessels are seen, but endothelial cells are specified and vessels are otherwise formed as normal. The most probable explanation for this stems from a fundamental difference in the regulation of *flk1* expression in the zebrafish. In mouse embryos, *flk1* is the first gene to be expressed in emerging haemangioblasts and endothelial precursors, initiating prior to and driving expression of *scl*. However, in zebrafish, *flk1* expression commences later, when haemangioblasts have already been specified, and is partially dependent on Scl. Although the existence of an unidentified earlier-acting VEGF receptor cannot yet be completely ruled out, it seems unlikely that VEGF plays such a critical role in endothelial specification in zebrafish.

So what might be driving endothelial specification in the zebrafish embryo? Ets factors are likely candidates. Gene expression is regulated by the binding of transcription factors to regulatory elements in the DNA, called enhancers, which promote transcription of the gene by RNA polymerase. One gene can have several different enhancers that serve to drive expression in different tissues and at different times during development. Ets factor binding sites in a *flk1* regulatory enhancer sequence have been shown, through mutagenesis, to be essential for *f k1* expression in endothelial cells in transgenic mice, and can be activated by coexpression with Ets1 and Ets2 [22]. Characterisation of a downstream enhancer that drives expression of *scl* in haematopoietic stem cells, endothelial cells, and haemangioblasts revealed critical GATA and Ets factor binding sites [23]. The same Ets-Ets- GATA motif was subsequently found to be present in candidate enhancers in the first introns of the Ets factor, *fli1*, and *hhex*—a transcription factor also expressed in haemangioblasts, in endothelial cells, and in the haematopoietic stem cell [24]. Expression of Lmo2, the partner of Scl, in endothelial cells has also recently been shown to be driven by its proximal promoter, which binds the Ets factors Elf1, Fli1, and Ets1 in vivo [25].

The Ets factors comprise a large multigene family of transcription factors. Previous attempts to identify Ets factors with important roles in zebrafish endothelial development have proven unsuccessful. Although the Ets factor *fli1* is one of the earliest genes to be expressed in haemangioblasts in the lateral plate mesoderm in zebrafish embryos, its loss-of-function phenotype, both in zebrafish and in mice, is relatively mild (A. Rodaway, N. Lawson, personal communication; [26]). In a recent study, knockdown of several zebrafish Ets factors, including *ets1*, *fli1*, *fli1b*, or *mef/elf4*, either alone or combined, failed to reveal any requirement for haematopoietic or endothelial development [27].

**Figure 3:**
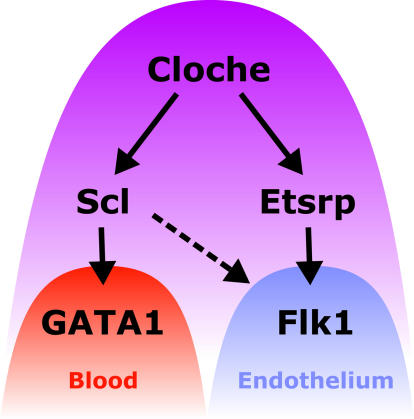
A Putative Model for the Regulation of Haemangioblast Specification and Differentiation in the Zebrafish Posterior Lateral Plate Mesoderm *Cloche* acts to specify the haemangioblast and is required for the expression of both *scl* and *etsrp* [5,29]. Scl is essential for the initiation of GATA*1* expression and for all subsequent haematopoietic development [30]. Although endothelial cells are formed in the absence of Scl, *flk1* expression is reduced from the outset, culminating in the failure of dorsal aorta specification [30]. Conversely, Etsrp is essential for *flk1* expression and for elaboration of the endothelial programme.

Therefore, it is of great interest that in this edition of *PLoS Biology*, Sumanas and colleagues [28] report the expression and functional characterisation of a novel Ets factor, Ets1-related protein (Etsrp), which is essential for endothelial development in zebrafish embryos. Crucially, this is the earliest identified gene in the hierarchy of transcription factors governing endothelial development that does not also affect haematopoietic development, unlike *cloche*, for example, and may therefore be involved in specification of endothelial cell fate from the haemangioblast. While *cloche* acts to specify the haemangioblast, Etsrp may be required in the subsequent decision—whether to become a blood cell or an endothelial cell (Figure 3).

## The Emphasis on Different Signals and Factors at Different Stages of Vascular Development May Vary between Species

Previous experiments in avian and amphibian embryos have suggested that fibroblast growth factor (FGF) signalling can induce angioblast formation in mesodermal tissues [31–33]. Although FGF signalling is known to be involved in angiogenesis [34], how the FGF pathway influences endothelial commitment is still unclear. Interestingly, many Ets factors have been shown to be targeted by the mitogen-activated protein kinase (MAPK) signalling pathway. Phosphorylation of different Ets factors by MAPK can affect DNA binding, transcriptional activity, protein stability, and subcellular localisation [35]. The MAPK signalling cascade is one of the major pathways that transmit FGF signals [36]. It is therefore possible that in the absence of VEGF signalling, FGF/MAPK signalling to Etsrp drives endothelial specification in zebrafish. Conceivably, different signalling pathways play more or less dominant roles during early development in different species. In this way, the players and set pieces are essentially the same, but the order of play can vary. For example, whereas VEGF signalling is important early in vasculogenesis in the mouse, it is only required at later stages in the zebrafish. The precise roles of individual Ets factors in the mouse may differ from those in the zebrafish, but an understanding of their functions in zebrafish will also likely inform our overall understanding of their roles in mammalian vascular development.

## Abbreviations

Etsrp: Ets1-related protein
FGF: fibroblast growth factor
MAPK: mitogen-activated protein kinase
VEGF: vascular endothelial growth factor
